# Supplementary material for: The IFIH1/MDA5 rs1990760 Gene Variant (946Thr) Differentiates Early- vs. Late-Onset Skin Disease and Increases the Risk of Arthritis in a Spanish Cohort of Psoriasis
Source: Int J Mol Sci. 2023 Sep 30;24(19):14803. doi: 10.3390/ijms241914803 (PMC10572774; doi:10.3390/ijms241914803)
Supplement: Supplementary file 1 [file ijms-24-14803-s001.zip › ijms-2447177-supplementary.pdf]

Suppl. Table S1. Distribution of the *IFIH1* genotypes according to the Cw6*0602 status.

		Psor ≤40	Psor >40		Cts ≤40 n=100	Cts >40 N=100	Cts total N=200
	**rs1990760**	N=211	N=30		N= 7	N =6	N=13
**Cw6 +ve**	TT	94 (45%)	8 (27%)	p=0.06	5	3	8 (62%)
	TC	91 (43%)	15 (50%)		1	2	3 (23%)
	CC	26 (12%)	7 (23%)		1	1	2 (15%)
		N=229	N=102		N=93	N=94	N=187
**Cw6 -ve**	TT	85 (37%)	32 (32%)	p<0.001	37	40	77 (41%)
	TC	129 (56%)	41 (40%)		42	42	84 (45%)
	CC	15 (7%)	29 (28%)		14	12	26 (14%)
	**rs35337543**						
**Cw6 +ve**	GG	210 (99.5%)	28 (94%)	p=0.05	7	6	13 (100%)
	GC	1 (0.5%)	2 (6%)		0	0	0
**Cw6 -ve**	GG	220 (96%)	100 (98%)	p=0.29	91	92	183 (98%)
	GC	9 (4%)	2 (2%)		2	2	4
	**rs35744605**						
**Cw6 +ve**	CC	210 (99.5%)	29 (97%)	p=0.23	7	6	13 (100%)
	CA	1 (0.5%)	1 (3%)		0	0	
**Cw6 -ve**	CC	222 (97%)	100 (98%)	p=0.44	92	92	184 (98%)
	CA	7 (3%)	2 (2%)		1	2	3 (2%)

Rs1990760 C>T

EUR	C: 0.395 (397)	T: 0.605 (609)	C|C: 0.141 (71)	C|T: 0.507 (255)	T|T: 0.352 (177)
CEU	C: 0.384 (76)	T: 0.616 (122)	C|C: 0.111 (11)	C|T: 0.545 (54)	T|T: 0.343 (34)
FIN	C: 0.409 (81)	T: 0.591 (117)	C|C: 0.162 (16)	C|T: 0.495 (49)	T|T: 0.343 (34)
GBR	C: 0.385 (70)	T: 0.615 (112)	C|C: 0.154 (14)	C|T: 0.462 (42)	T|T: 0.385 (35)
IBS	C: 0.355 (76)	T: 0.645 (138)	C|C: 0.131 (14)	C|T: 0.449 (48)	T|T: 0.421 (45)
TSI	C: 0.439 (94)	T: 0.561 (120)	C|C: 0.150 (16)	C|T: 0.579 (62)	T|T: 0.271 (29)

rs35337543 C>G

EUR	C: 0.981 (987)	G: 0.019 (19)	C|C: 0.962 (484)	C|G: 0.038 (19)
CEU	C: 0.990 (196)	G: 0.010 (2)	C|C: 0.980 (97)	C|G: 0.020 (2)
FIN	C: 1.000 (198)		C|C: 1.000 (99)	
GBR	C: 0.984 (179)	G: 0.016 (3)	C|C: 0.967 (88)	C|G: 0.033 (3)
IBS	C: 0.953 (204)	G: 0.047 (10)	C|C: 0.907 (97)	C|G: 0.093 (10)
TSI	C: 0.981 (210)	G: 0.019 (4)	C|C: 0.963 (103)	C|G: 0.037 (4)

Rs35744605 C>A

EUR	C: 0.996 (1002)	A: 0.004 (4)	C|C: 0.992 (499)	A|C: 0.008 (4)
CEU	C: 1.000 (198)		C|C: 1.000 (99)	
FIN	C: 0.995 (197)	A: 0.005 (1)	C|C: 0.990 (98)	A|C: 0.010 (1)
GBR	C: 0.989 (180)	A: 0.011 (2)	C|C: 0.978 (89)	A|C: 0.022 (2)
IBS	C: 0.995 (213)	A: 0.005 (1)	C|C: 0.991 (106)	A|C: 0.009 (1)
TSI	C: 1.000 (214)		C|C: 1.000 (107)	

Suppl. Figure S1. Allele and genotype frequencies for the three IFIH1 SNPs in different
European populations.

Eur: Europeans admixed.
CEU: Utah Residents of European ancestry.
FIN: Finnish in Finland.
GBR: British in England and Scotland.
IBS: Iberian population in Spain.
TSI: Toscani in Italy.
